# Extended-Spectrum β-Lactamase-Producing *Enterobacterales* in Free-Roaming Cats in Israel

**DOI:** 10.3390/ani16172736

**Published:** 2026-09-02

**Authors:** Neria Menashri, Tal Zilberman Daniels, Anat Shnaiderman-Torban, Danielle Keidar-Friedman, Sharon Tirosh-Levy, Talya Adler, Anat Lichter-Peled, Noor Shanir, Sharon Amit, Amir Steinman

**Affiliations:** 1 Koret School of Veterinary Medicine (KSVM), The Robert H. Smith Faculty of Agriculture, Food and Environment, The Hebrew University of Jerusalem, Rehovot 7610001, Israel; neriamenashri@gmail.com (N.M.); ashnaiderman@gmail.com (A.S.-T.); sharontirosh@gmail.com (S.T.-L.); talya.covrigaro@mail.huji.ac.il (T.A.); noor.shanir@mail.huji.ac.il (N.S.); 2 Clinical Microbiology, Sheba Medical Centre, Ramat-Gan 5266202, Israel; tal.zilbermandaniels@sheba.gov.il (T.Z.D.); sharon.amit@sheba.health.gov.il (S.A.); 3 Emerging Infectious Diseases Research Laboratory, Assuta Ashdod University Hospital, Ashdod 7747629, Israel; danielleke@ashmc.co.il; 4 Eshel Hanasi Veterinary Clinic, Eshel Hanasi Youth Village, Eshel Hanasi 8531000, Israel; lichter.peled@gmail.com; 5 Gray Faculty of Medical and Health Sciences, Tel-Aviv University, Tel-Aviv 6997801, Israel

**Keywords:** free-roaming cats, antimicrobial resistance, extended-spectrum β-lactamase (ESBL), companion animals

## Abstract

Amid the rising prevalence of antimicrobial resistance (AMR), attention is increasingly directed toward investigating additional factors that may contribute to or mediate its development, among which companion animals have emerged as a potential reservoir and transmission interface. Despite this, the contribution of free-roaming animal populations living in proximity to humans and domestic animals has yet to be thoroughly investigated worldwide. Accordingly, this study aimed to determine the prevalence and antimicrobial resistance patterns of extended-spectrum β-lactamase (ESBL)-producing *Enterobacterales* in free-roaming cats in Israel. Rectal swabs were collected from 214 free-roaming cats admitted to two veterinary clinics between 2023 and 2024. ESBL-PE were recovered from 51 cats (23.8%), yielding 61 isolates, which were tested for susceptibility to multiple antimicrobials. Eleven bacterial species were identified, with *Escherichia coli* being the most frequently recovered (62.3%, 38/61). Nine purposively selected *E. coli* isolates underwent whole-genome sequencing (WGS). Multidrug resistance (MDR), defined as non-susceptibility to at least one agent in three or more antimicrobial categories, was observed in 72.1% (44/61) of isolates, and four isolates were carbapenemase-producing *Enterobacterales* (CPE). These findings suggest that free-roaming cats may serve as a previously overlooked ‘One Health’ link, possibly connecting environmental contamination to human and animal health risks.

## Simple Summary

Over the last few decades, antimicrobial resistance has become a global health concern, rendering multiple antibiotics ineffective against common infections. Because antimicrobial resistance can spread across the human, animal and environmental sectors, it is best addressed through the holistic ‘One Health’ approach, a unified framework that enables monitoring of resistance trends and assessing the effectiveness of control strategies. While most research has historically focused on food-producing animals, and more recently on companion animals, synanthropic species that exist at the interface of human settlements and natural habitats have often been overlooked. Free-roaming cats, an animal population that inhabits the urban interface, where they frequently interact with humans, other domestic animals, and the environment, serve as an example of this oversight. To examine the role of these animals as vectors of dissemination, we assessed the antimicrobial susceptibility of extended-spectrum β-lactamase-producing *Enterobacterales* recovered from free-roaming cats in Israel. Our analysis revealed a high prevalence of both carriage and multidrug resistance within this population. Although further studies are needed to fully determine the contribution of free-roaming cat populations to antimicrobial dissemination, this study’s results demonstrate that they possibly act as an antimicrobial resistance reservoir for both humans and other species.

## 1. Introduction

Antimicrobial resistance (AMR) has become a global health concern over the last few decades, as it renders many antibiotics ineffective against infections that were once easily treatable [1,2]. As a worldwide threat with rapid geographic dissemination in diverse, interconnected systems, it stresses the need for a coordinated monitoring system to track resistance trends and evaluate the effectiveness of control strategies [3]. Among the most challenging resistance mechanisms are extended-spectrum β-lactamases (ESBL), which are increasingly detected in *Enterobacterales* across human, animal, and environmental sectors [4,5,6]. These enzymes can hydrolyze most first line β-lactam antibiotics, including third-generation cephalosporins, making the treatment of resulting infections particularly challenging. The genes encoding ESBLs are typically carried by mobile genetic elements, facilitating their rapid spread across a range of bacterial species and environments [7].

While many existing publications have historically focused on the prevalence and transmission dynamics of ESBLs in food-producing animals (e.g., cattle, poultry, and pigs) [8,9], their detection in companion animals is gaining increasing attention [10,11,12,13]. This widespread distribution is of particular concern because domestic companion animals (pets) were reported to carry ESBL-producing bacteria and, due to their close contact with humans, represent a potential reservoir for zoonotic transmission of these resistant pathogens into the household and community [14]. Molecular studies have since shown that the most prevalent ESBL-encoding genes in companion animals, particularly the CTX-M group, are highly related or even identical to those circulating in human clinical isolates, highlighting a clear genetic link for potential inter-species transmission [15,16,17]. Alarmingly, some research showed that the presence of carbapenemase-producing *Enterobacterales* (CPE) was detected, even though carbapenem antibiotics are reserved as a last-resort treatment and not authorized for routine veterinary use [18,19].

Free-roaming cats are socialized or unsocialized domestic cats (*Felis catus*) that are not consistently confined to an owner’s home or property and may spend time outdoors without direct supervision. This includes stray cats, which are generally defined as previously owned cats that became lost, escaped, or were abandoned, as well as their offspring [20]. Unlike household pets, free-roaming companion animal populations lack access to regulated, structured veterinary antibiotic stewardship and routine hygiene controls. When combined with their unrestricted movement in urban and semi-urban environments, it enables them to act as a dynamic bridge that facilitates the transfer of resistance genes between wildlife, domestic animals, contaminated environmental sources, and areas frequently occupied by humans [21,22,23,24]. Although free-roaming companion animals may play an important role in AMR ecology, they have been underrepresented in the literature until recently. Nonetheless, the available studies, among them those that focused on free-roaming cats, have yielded important insights into AMR patterns and have strengthened the hypothesis that multidrug resistant (MDR) *Enterobacterales* circulate within this specific animal population [25,26]. This study sought to further explore the potential of free-roaming cats to contribute to the spread of AMR in Israel, which is densely populated by free-roaming cats [27]. The data and results from this study may serve as a basis for the future development of effective risk assessment and control measures.

## 2. Materials and Methods

### 2.1. Animals and Sampling

This prospective study was carried out on two different cohorts. The first one consisted of unhealthy free-roaming cats that were admitted to the Koret School of Veterinary Medicine-Veterinary Teaching Hospital (KSVM-VTH) due to various health conditions. The second population comprised apparently healthy free-roaming cats, based on a standard physical examination performed by a veterinarian upon admission confirming the absence of overt clinical signs of disease, that were captured as part of Trap–Neuter–Return (TNR) procedures coordinated by the Eshel Hanasi Veterinary Clinic (EHVC). Rectal swab samples were collected from the cats within 24 h of arrival and transferred in solid AMIES media (Meus s.r.l., Piove di Sacco, Italy) for laboratory processing and *Enterobacterales* recovery. The study was approved by the Hebrew University Ethics Committee (PET-2023-12-NE).

### 2.2. Enterobacterales Isolation and Identification

Sample processing was performed by a single operator, under similar conditions, following standard protocol used in previous studies [28]. Upon arrival at the laboratory, all samples were inoculated directly into unsupplemented Brain-Heart Infusion (BHI) enrichment broth (HyLabs, Rehovot, Israel) without selective antibiotics. This non-selective enrichment step was employed to resuscitate stressed or low-abundance bacteria from the primary sample prior to selective isolation, in accordance with established clinical microbiology protocols to increase detection sensitivity [29]. After incubation at 37 °C for 24 h, enriched samples were plated onto CHROMagar ESBL plates (HyLabs, Rehovot, Israel) and incubated for an additional 24 h at 37 °C. Colonies that appeared after overnight incubation were recorded, and up to three colonies of each distinct color and morphology were re-streaked onto fresh plates to obtain pure bacterial cultures. The bacterial isolates were identified by matrix-assisted laser desorption/ionization time-of-flight mass spectrometry (MALDI-TOF, Microflex LT, Bruker Daltonics, Billerica, MA, USA). Pure isolates were stored at −80 °C until further analysis.

### 2.3. Antimicrobial Susceptibility Testing

All testing was performed in an ISO 15189-accredited clinical microbiology laboratory. ESBL production was confirmed in all *Enterobacterales* isolates using a positive disc approximation test based on the synergy between clavulanate and several different β-lactam subclasses, which were interpreted in accordance with CLSI M100 (2022) human breakpoints. After overnight incubation at 37 °C, isolates showing an increased zone of inhibition around the amoxicillin-clavulanic acid disc (the “keyhole” effect) were identified as ESBL producers. For equivocal results, a combination disc test with cefotaxime and ceftazidime, both with and without clavulanic acid, was used. An increase of ≥5 mm in the inhibition zone diameter with clavulanic acid compared with cephalosporins alone confirmed ESBL production. For isolates exhibiting carbapenem resistance, the lateral flow immunoassay Carba-5 (NG-Biotech, Guipry, France) was performed according to the manufacturer’s instructions to identify specific carbapenemase types [30]. Isolates exhibiting phenotypic non-susceptibility to at least one carbapenem (ertapenem or meropenem) were categorized as Carbapenem-Resistant *Enterobacterales* (CRE). Isolates confirmed to produce carbapenemase enzymes via lateral flow immunoassay were categorized as Carbapenemase-Producing *Enterobacterales* (CPE). A complete antibiogram was later determined using the Vitek-2 system (Biomerieux Inc., Marcy l’Etoile, France) with the AST-N395 card according to manufacturer’s recommendations and CLSI guidelines, accounting for species-specific intrinsic resistance. Tested agents included ampicillin, amoxicillin-clavulanic acid, piperacillin/tazobactam, cefazolin, cefuroxime, cefuroxime axetil, (not a released result), ceftazidime, ceftriaxone, ertapenem, meropenem, amikacin, gentamicin, ciprofloxacin, fosfomycin (urine only in clinical use), nitrofurantoin (urine only in clinical use), chloramphenicol and trimethoprim/sulfamethoxazole. Quality control was routinely performed per CLSI requirements using standard reference strains (*E. coli* ATCC 25922 and *E. coli* ATCC 35218). Intermediate and resistant categories were grouped as non-susceptible. MDR was defined as non-susceptibility to at least one agent in three or more antimicrobial categories [31].

### 2.4. Whole Genome Sequencing Analysis of ESBL-PE Isolates

#### 2.4.1. DNA Extraction, Whole Genome Sequencing and Analysis

A total of nine *E. coli* isolates were selected for whole-genome sequencing (WGS) based on their phenotypic diversity across antimicrobial non-susceptibility profiles. Prior to DNA extraction, stored isolates were grown overnight on CHROMagar medium (HyLabs, Rehovot, Israel). Extraction was performed using the MagCore^®^ Super automated extraction system (RBC Bioscience^®^, New Taipei City, Taiwan), following the manufacturer’s protocol for the MagCore^®^401 Genomic DNA Tissue Kit. DNA concentration and purity were tested using DeNovix DS-11 FX+ spectrophotometer (DeNovix, Wilmington, DE, USA). DNA libraries were prepared using the NEBNext^®^ Ultra™ II FS DNA Library Prep Kit for Illumina (New England Biolabs, Ipswich, MA, USA). Library quantification and quality control were performed using the Qubit dsDNA High Sensitivity Assay Kit (Thermo Fisher Scientific, Waltham, MA, USA) on a Qubit 2.0 Fluorometer. Library size distribution was subsequently assessed using a High Sensitivity D5000 ScreenTape with the TapeStation 2200 System (Agilent Technologies, Santa Clara, CA, USA). Finally, sequencing was performed on the NextSeq 1000 platform (Illumina, San Diego, CA, USA) in accordance with the manufacturer’s recommendations, utilizing the NextSeq™ 1000/2000 P1 XLEAP-SBS™ Reagent Kit (600 Cycles) with a target of 840,000 reads per sample.

FastQC v0.12.1 software was used for the assessment of read quality (https://www.bioinformatics.babraham.ac.uk/projects/fastqc/ accessed on 14 March 2026). Trimmomatic v.0.39 was used for read trimming and removal of low-quality reads [32]. SPAdes genome assembler v4.0.0 was used for de novo assembly of paired-end reads [33]. Assemblies were analyzed using Checkm2 for contamination [34]. Isolates were initially assigned to sequence types using conventional Seven-gene multi-locus sequence typing (MLST) according to the Achtman scheme via the PubMLST database (https://pubmlst.org/ accessed on 14 March 2026) [35,36]. Antimicrobial resistance genes and mutations were identified using AMRFinderPlus v3.12.8 [37]. Plasmid replicons were annotated using PlasmidFinder [38]. Higher-resolution genomic relationships among isolates representing the identified STs were then investigated using core-genome MLST (cgMLST) analysis implemented in chewBBACA v3.4.2 [39], with relatedness assessed according to pairwise allelic differences across core-genome loci, and results were visualized using GrapeTree v2.2 [40]. Genome annotation was performed using Prokka v1.14.6 [41].

#### 2.4.2. Phylogenetic Analysis

For core genome phylogenetic analysis of the isolates, pan-genome determination was performed using Panaroo v1.5.2 [42]. Core gene alignment was processed using Gubbins v3.3.1 [43] to remove variants that are the result of recombination events. Additional genome assemblies of each ST were searched for in BakRep [44] (https://bakrep.computational.bio/ accessed on 14 March 2026) and retrieved from NCBI GenBank database. For ST-specific phylogenetic analysis, we used Snippy (https://github.com/tseemann/snippy/ accessed on 14 March 2026) v.4.6.0, followed by snp-dists (https://github.com/tseemann/snp-dists/ accessed on 14 March 2026) to assess core SNP distance. The following genomes were used as references in the Snippy analyses: ST38: GCA_024917375.1, ST155: GCA_022180165.1, ST1431: GCA_900607365.1. Maximum likelihood phylogenetic trees were constructed using IQ-TREE v.2.4.0 [45] with ModelFinder for automatic model selection and 1000 ultrafast bootstrap replicates [46]. Trees were visualized and annotated using iTOL v6 (https://itol.embl.de/ accessed on 14 March 2026).

### 2.5. Sample Size and Statistical Analysis

The minimum sample size for free-roaming cats was calculated using WinPepi (version 11.65), based on an estimated prevalence of 12.8% for ESBL-producing *Escherichia coli* (derived from a previous study investigating *E. coli* carriage [47]), assuming a 95% confidence level and a 5% acceptable margin of error. This yielded a required minimum sample size of n = 172. Due to the limited number of studies available in this area, the sample size calculation was based on the prevalence estimate available to us, namely the ESBL-producing *E. coli* prevalence reported in a previous study [47]. Since our study examined *Enterobacterales* as a broader group, we anticipated that the overall prevalence would be higher than that reported for *E. coli* alone, while still expecting *E. coli* to remain the predominant species. Therefore, we deliberately used a larger sample size (n = 214) than the minimum required for the *E. coli*-based estimate to better accommodate the expected higher prevalence across *Enterobacterales*.

Associations between ESBL-PE carriage and host/population factors (including health status, age group, and sex) were evaluated, alongside differences in bacterial species distribution and antimicrobial resistance profiles among recovered isolates. Prevalence estimates are presented as percentages with corresponding 95% confidence intervals (CIs). Missing values were excluded solely from the relevant analysis. Specifically, age records were missing for 10 cats (4.7% of the cohort, all originating from the unhealthy cohort), restricting the age-specific analytical sample to n = 204 (95.3% of the total cohort).

Differences in prevalence between groups were assessed using Pearson’s chi-square test or Fisher’s exact test, as appropriate. All statistical analyses were conducted using SPSS for Mac, version 29.0 (SPSS Inc., Chicago, IL, USA). Statistical significance was defined as a *p*-value < 0.05.

## 3. Results

### 3.1. Cat Population

A total of 214 free-roaming cats were sampled during this study across two distinct cohorts at geographically separate veterinary centers, including 47.2% (101/214) that were apparently healthy and admitted as part of TNR programs to the EHVC. The remaining 113 cats (52.8%) were admitted to the KSVM-VTH due to various health conditions, including general trauma or infectious diseases. Males accounted for 52.3% (112/214) of the total study population. Among cats with documented age, 30 (14.7%) were classified as Young (<2 years) and 174 (85.3%) as Adult (≥2 years) (Table 1).

### 3.2. Prevalence of ESBL-PE Isolates

**Figure 1:**
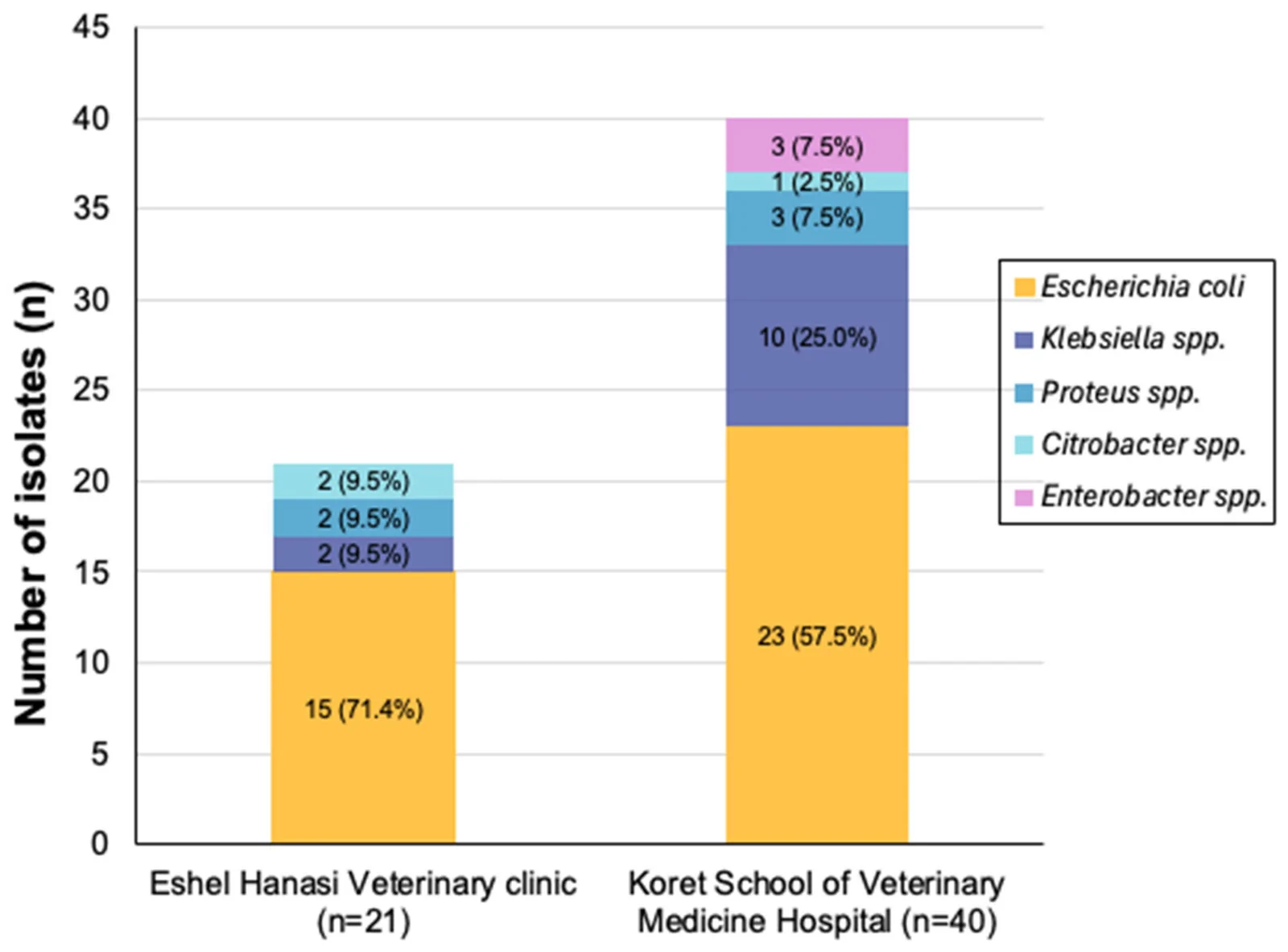
Species distribution of ESBL-PE isolates recovered from the two cohorts: unhealthy cats admitted to the Koret School of Veterinary Medicine-Veterinary Teaching Hospital (KSVM-VTH; n = 40 isolates), and apparently healthy cats sampled during Trap–Neuter–Return (TNR) procedures at the Eshel Hanasi Veterinary Clinic (EHVC; n = 21 isolates).

Among the 214 cats sampled in this study, 51 (23.8%, 95% CI: 18.3–30.1%) were identified as ESBL-PE carriers. The prevalence of carriage was notably higher among unhealthy cats at 31.0% (35/113, 95% CI: 22.6–40.4%) than among apparently healthy cats (15.8%, 16/101, 95% CI: 9.3–24.4%; *p* = 0.009). From the 51 carriers, a total of 61 isolates were recovered. The majority of these isolates, 65.6% (40/61), were obtained from the 35 unhealthy cats, while approximately one-third, 34.4% (21/61), were recovered from the 16 apparently healthy cats. Certain individuals were co-colonized with multiple resistant strains, contributing to the overall diversity of the recovered ESBL-PE isolates. Eleven bacterial species were identified, of which four were identified in both apparently healthy and unhealthy cats—*E. coli*, *Klebsiella pneumoniae*, *Proteus penneri* and *Citrobacter freundii. E. coli* was the predominant species, accounting for 62.3% (38/61) of the total isolates, followed by *K. pneumoniae* at 18% (11/61). The remaining ESBL-PE included other *Proteus* (5/61), *Enterobacter* (3/61), *Citrobacter* (3/61) and *Klebsiella* species (1/61) (Figure 1).

### 3.3. Antimicrobial Susceptibility Rates and Multidrug Resistance (MDR)

**Figure 2:**
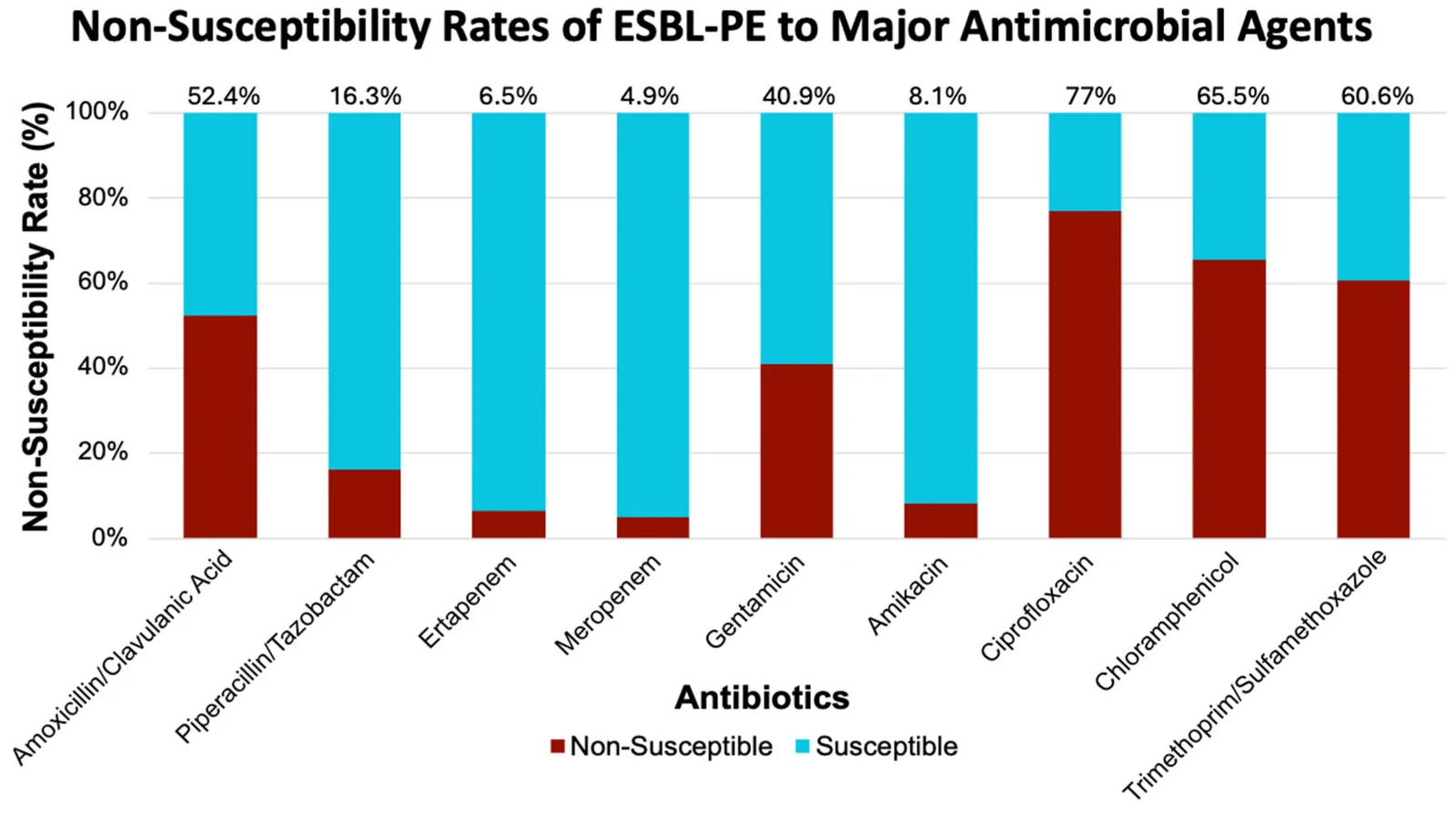
Antimicrobial non-susceptibility profiles of ESBL-PE isolates recovered from free-roaming cats in the present study.

Among the 61 ESBL-PE isolates, non-susceptibility to non-β-lactam agents was common, particularly to ciprofloxacin (77.0%, 47/61, 95% CI: 64.5–86.8%), chloramphenicol (65.5%, 40/61, 95% CI: 52.3–77.2%), and trimethoprim/sulfamethoxazole (60.6%, 37/61, 95% CI: 47.3–72.9%) (Figure 2). Aminoglycoside non-susceptibility was also observed, more frequently for gentamicin (40.9%, 25/61, 95% CI: 28.5–54.3%) than for amikacin (8.1%, 5/61, 95% CI: 2.7–18.1%). Among β-lactam/β-lactamase inhibitor combinations, 52.4% (32/61, 95% CI: 39.2–65.4%) of isolates were non-susceptible to amoxicillin/clavulanic acid and 16.3% (10/61, 95% CI: 8.2–28%) to piperacillin/tazobactam. Carbapenem non-susceptibility was less frequent, with 6.5% (4/61, 95% CI: 1.8–15.9%) non-susceptible to ertapenem and 4.9% (3/61, 95% CI: 1–13.7%) to meropenem. When ESBL-mediated resistance to penicillins and cephalosporins was considered as a single β-lactam resistance category, 72.1% (44/61) of isolates met the definition of multidrug resistance, defined as non-susceptibility to at least one agent in three or more antimicrobial categories [30]. Four isolates were classified as carbapenem-resistant *Enterobacterales* (CRE); all four CRE isolates were confirmed to be carbapenemase-producing *Enterobacterales* (CPE) via lateral flow immunoassay, comprising three NDM producers and one OXA-48-like carbapenemase producer (Table S1).

### 3.4. Genomic Characterization and MLST of Selected ESBL-Producing E. coli Isolates

**Figure 3:**
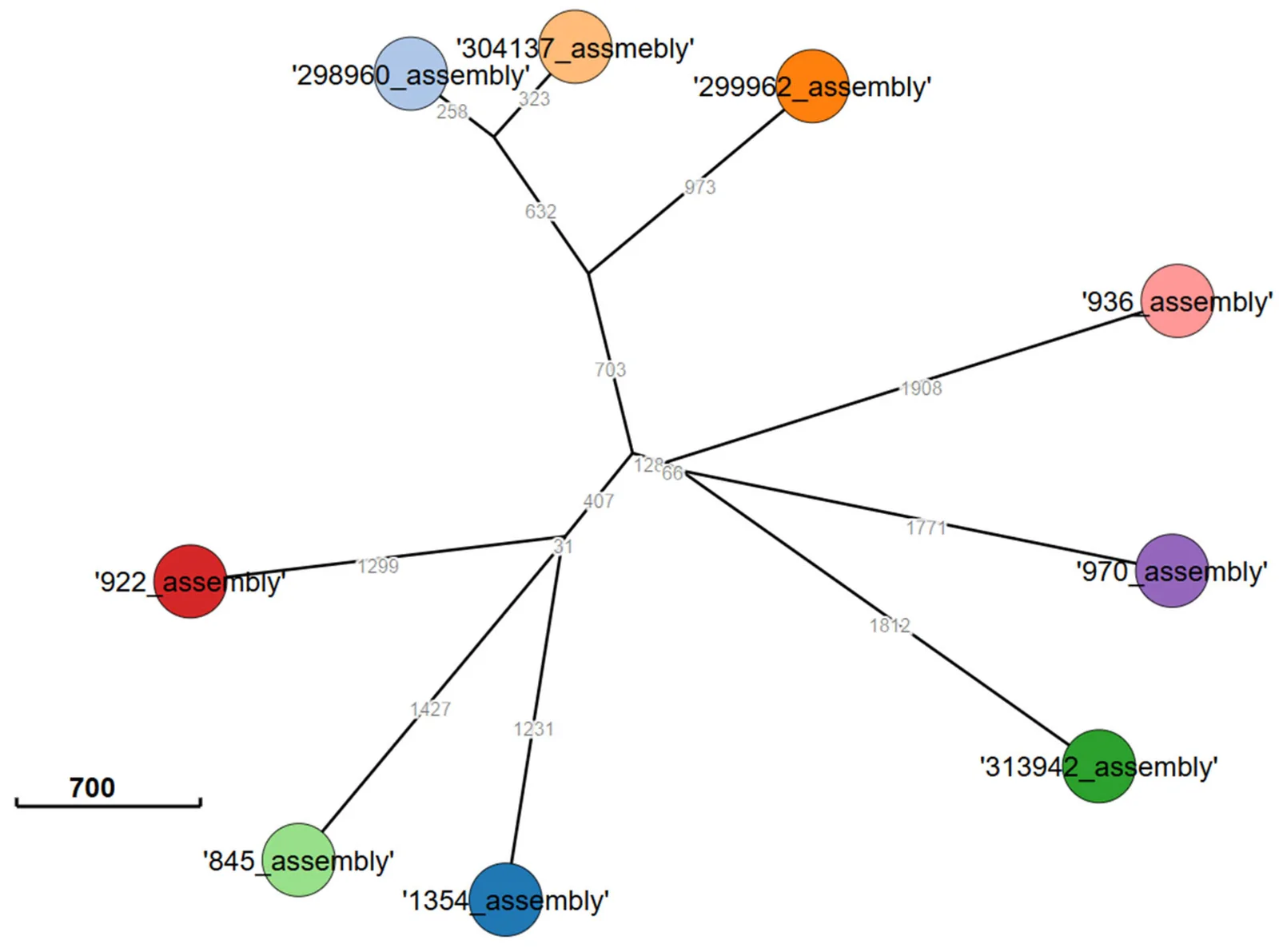
Minimum spanning tree (MST) based on cgMLST allele profiles (generated using chewBBACA). Each node represents one isolate. Edges connect isolates with minimum total allelic distance to span all nodes; edge lengths or labels relate to the number of differing alleles between profiles.

We selected nine *E. coli* isolates with diverse resistance phenotypes for WGS (Table S2). In silico MLST revealed a high degree of clonal diversity, as each isolate belonged to a distinct sequence type (ST). This heterogeneity was further supported by the cgMLST-based phylogenetic tree (Figure 3), which showed significant genetic distances between isolates. Although isolates 304137 and 298960 formed a proximal cluster, all other isolates were highly divergent, characterized by extended branch lengths that indicate the circulation of distinct, unrelated resistant lineages within this study’s free-roaming cat population.

**Figure 4:**
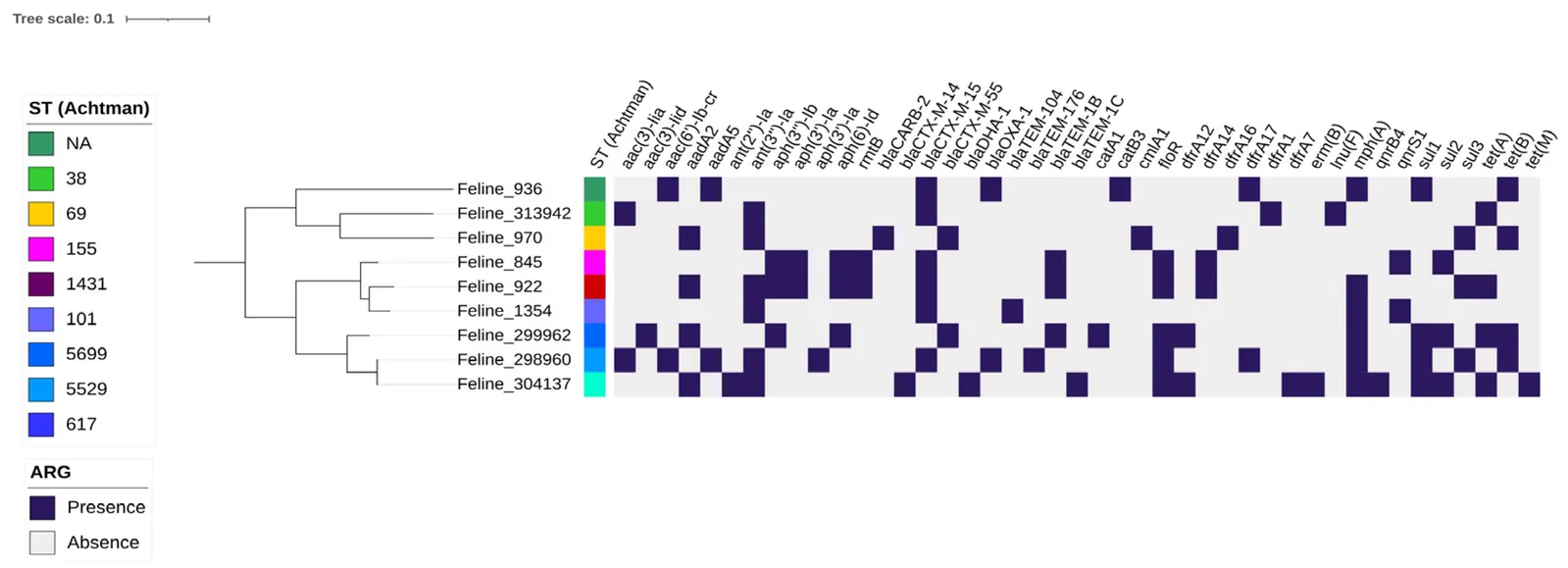
Maximum likelihood phylogenetic tree of the nine *E. coli* genome assemblies, based on core genome single nucleotide polymorphism (SNP), with their antibiotic resistance gene (ARG) profile.

As mentioned above, the antibiograms of the ESBL-PE demonstrated significant resistance to other classes of antimicrobials, suggesting additional resistance mechanisms beyond ESBL production. We therefore analyzed the full genome for antibiotic resistance genes (ARGs) (Figure 4). While the nine *E. coli* isolates harbored *bla*CTX-M variants (14/15/55), they presented different combinations of ARGs such as other β-lactamases (*bla*DHA-1, *bla*TEM), as well as recognized ARGs to all major antibiotic classes: aminoglycosides, fluoroquinolones, tetracyclines, macrolides and folate pathway inhibitors (Figure 3). No two isolates shared an identical ARG profile, and no single ARG variant was present in all isolates, indicating considerable heterogeneity between the resistome of the different STs. In addition, isolates carried different combinations of plasmid replicon types (for example, IncFIB, IncFIC, IncX1, Col, p0111).

To place the feline *E. coli* isolates in a broader genomic framework, ST-specific core genome phylogenies were reconstructed using additional assemblies from NCBI GenBank. Phylogenomic analysis revealed notable regional connections for specific isolates. Our analysis highlighted key local connections: the ST38 feline isolate (Feline_313942) clustered closely with human clinical isolates from Israel (pairwise distance: 47–98 core SNPs), alongside assemblies from the USA and UK, indicating a potential local human–animal interface (Figure S1). For ST155, the phylogeny placed our isolate Feline_845 in a cluster with isolates from Japan (2017) and the USA (1979), with pairwise core SNP distances of 107–133 (Figure S2). For ST1431, our isolate Feline_922 grouped within a diverse clade containing isolates from multiple continents and from human clinical, animal and environmental sources (Figure S3). Within this cluster, Feline_922 differed from the other ST1431 isolates by 47–129 core SNPs. For the rarer ST5529, a human clinical isolate from Lebanon was identified as the closest available genomic comparator, although it remained relatively divergent at 330 core SNPs. Finally, ST5699 showed greater genetic distances from available international comparator genomes, suggesting very distant phylogenetic relationships.

### 3.5. Association Between Cat Health Status and ESBL-PE Carriage

Statistical analysis (Table 1) revealed that the cat health status was significantly associated with carriage of ESBL-producing bacteria (OR = 2.38; 95% CI: 1.22–4.64; *p* = 0.009). Unhealthy cats sampled at the KSVM-VTH clinic were more than twice as likely to carry ESBLs as those from apparently healthy cats admitted as part of TNR programs to the EHVC. In contrast, no significant associations were found for sex (*p* = 0.921) or approximate age (*p* = 0.911), indicating that these demographic factors did not influence the prevalence of ESBL carriage in the study population.

## 4. Discussion

The present study identified ESBL-PE carriage in 23.8% (51/214) of the sampled free-roaming cat cohort. ESBL-producing *E. coli* was the predominant species among the recovered isolates, accounting for 62.3% (38/61), followed by *K. pneumoniae* and additional *Enterobacterales* species. Cross-study comparisons should be interpreted with caution, owing to differences in study populations, health status, sampling designs, microbiological methods, and whether estimates refer to overall ESBL-PE carriage or specifically to ESBL-producing *E. coli*. While past research focusing solely on feline ESBL-PE worldwide is somewhat limited, overall carriage rates are typically reported at <12.5% [12,48,49,50,51,52]. In comparison, our findings reveal a notably higher baseline, even when compared to prior local data. Specifically, a previous report from Israel documented a 16.9% ESBL prevalence exclusively among hospitalized, diseased cats [53]. Remarkably, the healthy cohort in our study exhibited a carriage rate closer to that previous report for sick cats (15.8%), while our diseased cohort reached a substantially higher prevalence of 31.0%. This comparison highlights a somewhat concerning escalation in ESBL-PE carriage within both apparently healthy and diseased feline populations in the region.

Likewise, the prevalence of ESBL-producing *E. coli* in our cohort (17.8%, found in 38/214 of our sampled cats) substantially exceeds the estimated global prevalence of cats ESBL-producing *E. coli*, which stands at 5.04% [54]. This pronounced burden is consistent with its central role as a critical sentinel organism for monitoring antimicrobial resistance at the human–animal–environment interface. These elevated rates observed here are also compatible with broader epidemiological observations indicating that the Mediterranean basin and neighboring Middle Eastern regions tend to have a greater burden of ESBL-PE carriage than many Western settings, in both human and veterinary populations [55,56]. In this context, our current overall feline ESBL-producing *E. coli* carriage rate (17.8%) exceeds regional reports from Italy (6.7–12.8%) [47,57] Saudi Arabia (7.1%) [58] and Portugal (7.8%) [58]. When accounting for health status, our findings show a continuous or slightly elevated trend in Israel, with the current carriage rate among diseased cats (20.3%, 23/113), similar to the prevalence among diarrheic cats in the Netherlands (25%, 5/20) and exceeding the 4.1% reported from diseased cats in Saudi Arabia [58], and the 8.1% documented in Portugal [59].

The high prevalence observed in this study may reflect a broader ‘One Health’ challenge, in which free-roaming cats serve as indicators, and potentially participants, in the circulation of resistant *Enterobacterales* within shared urban environments, as has been suggested in other animal species sharing these settings [60,61]. Compared with household pets, free-roaming cats generally do not benefit from the same level of veterinary care, oversight antimicrobial use and routine hygiene practices. They also move freely between human settlements, animal populations, refuse sites, and other environmental niches. These conditions may expose them to urban waste, untreated sewage, discarded human food, raw meat and contaminated environmental sources, all of which have been described as potential reservoirs of MDR *Enterobacterales* [62,63,64]. This challenge may be particularly relevant in Israel, where dense free-roaming cat populations are common in urban centers [27]. The convergence of high regional ESBL endemicity, dense urban free-roaming cat populations, and repeated exposure to human-associated environments may therefore help explain the substantial carriage rate observed in this cohort.

Carriage was not evenly distributed across the two sampled populations. ESBL-PE carriage was significantly higher among unhealthy cats admitted to KSVM-VTH than among apparently healthy cats sampled through TNR procedures at EHVC (Table 1). Because samples were collected within 24 h of admission, these findings are more consistent with pre-existing carriage than with acquisition during the current hospitalization. However, this association should not be attributed to health status alone. Although both cohorts consisted of free-roaming cats, those referred for veterinary care were more likely to have had prior contact with caregivers or municipal or primary veterinary services [65]. This interaction presents a plausible scenario for potential, undocumented antimicrobial exposure, which is a recognized risk factor for the selection of ESBL-producing organisms [66]. The two cohorts therefore differed not only clinically but also in their likely histories of human contact, antimicrobial exposure and catchment areas. Each of these factors may impose antimicrobial pressure or increase contact with resistant organisms. In contrast, the apparently healthy TNR cohort may represent a population with fewer medical interventions and potentially lower exposure to these selection pressures. Thus, the higher carriage observed among unhealthy cats likely reflects a combination of host conditions, prior healthcare or caregiver contact, antimicrobial exposure, and environmental pressures rather than a single explanatory factor.

The phenotypic resistance profile further supports the interpretation that free-roaming cats may harbor clinically relevant MDR *Enterobacterales*. Overall, 72.1% of isolates met the criteria for MDR, defined as non-susceptibility to at least one agent in three or more antimicrobial categories [31]. Grouping intermediate and resistant isolates into a single ‘non-susceptible’ category reflects clinical utility and aligns with standard reporting [30], though it may conservatively estimate resistance rates. Nevertheless, intermediate isolates accounted for only a minor fraction of our dataset, thereby minimizing any potential impact on overall MDR estimates. Non-susceptibility extended beyond ESBL-mediated β-lactam resistance and included high rates of non-susceptibility to ciprofloxacin, chloramphenicol, and trimethoprim/sulfamethoxazole, as well as resistance to aminoglycosides and β-lactam/β-lactamase inhibitor combinations (Figure 2). This broad co-resistance pattern is important because it suggests that ESBL-PE carriage in free-roaming cats is not limited to a single resistance mechanism, but frequently occurs within a wider multidrug-resistant background. The detection of four CRE isolates is particularly notable. Although carbapenem non-susceptibility remained uncommon relative to other antimicrobial classes, the presence of carbapenemase-producing *Enterobacterales* in a free-roaming cat population is concerning because carbapenems are advanced-line agents in human medicine and are not expected to be routinely used in this animal population [67]. This finding therefore raises the possibility of acquisition from human-associated or environmentally contaminated sources. Differences in regulatory and enforcement frameworks for critical antimicrobial use may further shape the emergence and dissemination of such resistance, particularly when contrasted with the restrictions applied in many European settings [18].

Genomic analysis of selected ESBL-producing *E. coli* isolates provided additional resolution and supported the phenotypic findings. The nine sequenced isolates showed substantial clonal diversity, with each isolate assigned to a distinct sequence type. This indicates that ESBL carriage in this cohort was not driven by a single expanding feline clone, but rather by multiple unrelated *E. coli* lineages circulating within the free-roaming cat population. Several of the detected sequence types, including ST101, ST617, ST69, and ST38, have previously been reported in human colonization studies as well as in clinical infections in humans and companion animals worldwide [68,69,70,71,72,73,74]. These findings do not prove direct transmission between cats and humans, but they do show that free-roaming cats may carry lineages that are also recognized in human and companion-animal contexts.

The broader phylogenomic analysis further supports the relevance of the human–animal–environment interface. The feline ST38 isolate Feline_313942 clustered most closely with human clinical isolates from Israel, differing by 47–98 core SNPs, suggesting a possible local epidemiological connection. For the ST5529 isolate, the closest available comparator was a human clinical isolate from Lebanon [75], although the distance of 330 core SNPs indicates a more distant relationship. Other sequence types, including ST155, ST5529, and ST5699, were more distantly related to available international comparator genomes. Taken together, these findings suggest that at least some resistant *E. coli* lineages found in free-roaming cats overlap phylogenetically with lineages detected in humans, while others reflect broader environmental or animal-associated diversity. This pattern is consistent with a ‘One Health’ model in which resistant bacteria and resistance genes circulate through multiple interconnected reservoirs, rather than through a simple unidirectional transmission pathway [70,76,77,78,79,80].

The resistome analysis was similarly heterogeneous. All nine sequenced *E. coli* isolates harbored *bla*CTX-M variants, specifically *bla*CTX-M-14, *bla*CTX-M-15, and *bla*CTX-M-55, consistent with their ESBL phenotype. In addition, the isolates carried diverse combinations of other β-lactamase genes, including *bla*TEM, *bla*OXA-1, *bla*CARB-2, and the plasmid-mediated AmpC β-lactamase gene *bla*DHA-1 (Figure 4). Co-resistance determinants associated with aminoglycosides, fluoroquinolones, trimethoprim/sulfamethoxazole, tetracyclines, and phenicols were also detected, in keeping with the broad phenotypic resistance observed. No two isolates shared an identical ARG profile, and no single resistance-gene variant was present in all isolates beyond the general presence of CTX-M-type ESBL genes. The presence of these determinants alongside diverse plasmid replicon types, including IncX1, IncN, and IncHI2, highlights the potential for horizontal gene transfer and persistence of multidrug resistance within the shared urban ecosystem [26,81].

This study has several limitations. First, sampling was concentrated in only two centers, which may introduce geographic and population-selection bias. Consequently, the findings may not fully reflect ESBL-PE carriage among free-roaming cat populations in Israel, particularly in more rural or isolated areas. Second, the two study cohorts differed in health status and source population, making it difficult to disentangle the effects of clinical condition, geography, and degree of human contact. Third, because free-roaming cats are unowned, detailed medical histories and records of previous antimicrobial treatment were unavailable. Cats referred for veterinary care may have had prior contact with caregivers or veterinary services and, consequently, undocumented antimicrobial exposure before sampling. Although carbapenems are not authorized for routine veterinary use in the region, such exposure, as well as acquisition of resistant organisms from human-associated or environmental sources, cannot be excluded. Fourth, owing to the limited availability of broader data, sample size calculations were based on previous studies of *E. coli.* As carriage across all *Enterobacterales* was expected to be higher, the cohort was intentionally expanded to 214 cats, exceeding the calculated minimum of 172. Fifth, age data were missing exclusively from the clinical cohort; however, as this affected only 10 cats, representing 4.7% of the total cohort, its impact on age-related analyses is likely minimal, although a minor non-random missingness pattern should be noted. Sixth, genomic analysis was limited to a selected subset of *E. coli* isolates and may therefore not capture the full diversity of ESBL-PE species recovered. Finally, the cross-sectional design provide a point-prevalence estimate and cannot account for temporal variation, duration of carriage, or directionality of transmission. Longitudinal studies incorporating simultaneous sampling of free-roaming cats, owned animals, humans, and environmental sources will be needed to delineate transmission pathways and clarify the role of free-roaming cats within the broader AMR ecosystem.

## 5. Conclusions

This study demonstrates a high prevalence of ESBL-producing *Enterobacterales* among free-roaming cats in Israel, with frequent multidrug resistance and the detection of carbapenemase-producing isolates. Carriage was particularly common among unhealthy cats, suggesting that previous medical exposure, antimicrobial pressure, caregiver or veterinary contact and environmental conditions may all contribute to the accumulation of resistant *Enterobacterales* in this population. Genomic analysis of selected *E. coli* isolates revealed diverse lineages, heterogeneous resistance-gene profiles, CTX-M-type ESBL determinants, and plasmid replicons associated with horizontal gene transfer. Importantly, our findings demonstrate bacterial carriage and colonization rather than active transmission. While these results support the view that free-roaming cats are part of the ‘One Health’ antimicrobial-resistance interface as relevant sentinels and potential environmental reservoirs, they do not establish direct transmission to humans or other species. Continued surveillance, combined animal–human–environment sampling, and integration of free-roaming animal populations into AMR risk assessment are needed to better define their role in the maintenance and dissemination of clinically important resistance.

## Figures and Tables

**Table 1:** Cats’ characteristics and their association with ESBL carriage in this study.

Variable	Category	Total, n (%)	ESBL-Positive (n)	Prevalence (%)	Odds Ratio (OR)	95% CI (OR)	*p* Value
Age							0.911
	Adult (≥2 years)	174 (85.3%)	39	22.4	(Ref)		
	Young (<2 years)	30 (14.7%)	7	23.3	1.05	0.35–2.78	
Sex							0.921
	Male	112 (52.3%)	27	24.1	(Ref)		
	Female	102 (47.7%)	24	23.5	0.97	0.49–1.91	
Health status							0.009 *
	Apparently healthy cats (EHVC)	101 (47.2%)	16	15.8	(Ref)		
	Unhealthy cats (KSVM-VTH)	113 (52.8%)	35	31.0	2.38	1.17–4.98	

* *p* < 0.05, indicating a statistically significant association.

## Data Availability

All genome drafts have been deposited in NCBI GenBank under BioProject PRJNA1429367.

## Supplementary Materials

The following supporting information can be downloaded at https://www.mdpi.com/article/10.3390/ani16172736/s1, Table S1: Detailed characterization of the for *E. coli* carbapenem-resistant isolates found in this study; Table S2: Genomic characteristics, sequence types, antibiotic resistance gene (ARG) profiles, and plasmid replicon types of the nine whole-genome sequenced *Escherichia coli* isolates; Figure S1: Maximum likelihood phylogenetic tree of ST38 genome assemblies based on core genome single nucleotide polymorphisms (SNPs). Tip labels are annotated with source, country and collection year. The tree is midpoint-rooted. Bootstrap support values >90 are indicated by purple stars. For ST155, the phylogeny placed our isolate Feline_845 in a cluster with isolates from Japan (2017) and the USA (1979), with pairwise core SNP distances of 107–133; Figure S2: Maximum likelihood phylogenetic tree of ST155 genome assemblies based on core genome SNPs, annotated with country and sample source. The tree is midpoint-rooted. Bootstrap support values > 90 are indicated by purple stars; Figure S3: Maximum likelihood phylogenetic tree of the ST1431 genome assemblies, based on core genome single nucleotide polymorphism (SNP), with sample source, country and collection year. Tree was rooted at midpoint. Bootstrap values >90 are presented by purple stars.

## Abbreviations

The following abbreviations are used in this manuscript:

AMR	Antimicrobial resistance
ARG	Antibiotic Resistance Gene
ESBL	extended-spectrum β-lactamase
CRE	Carbapenem-resistant *Enterobacterales*
CPE	Carbapenemase-Producing *Enterobacterales*
MDR	Multidrug Resistance
MLST	Multi-Locus Sequence Typing
WGS	Whole-Genome Sequencing
EHVC	Eshel Hanasi Veterinary Clinic
KSVM-VTH	Koret School of Veterinary Medicine-Veterinary Teaching Hospital
TNR	Trap–Neuter–Return
