# Supplementary material for: Extended-Spectrum β-Lactamase-Producing Enterobacterales in Free-Roaming Cats in Israel
Source: Animals (Basel). 2026 Sep 2;16(17):2736. doi: 10.3390/ani16172736 (PMC13564658; doi:10.3390/ani16172736)
Supplement: Supplementary file 1 [file animals-16-02736-s001.zip › animals-4456491-supplementary.pdf]

Supplementary Materials:

Table S1. Detailed characterization of the for *E. coli* carbapenem-resistant isolates found in this study.

Isolate ID	Origin	Species	Carbapenemase Gene	Minimum Inhibitory Concentration (MIC, µg/mL) and Interpretation																
				AMP	AMC	TZP	CFZ	CXM	FOX	CAZ	CTX	ETP	MEM	AMK	GEN	CIP	FOS	NIT	CHL	SXT
293203	KSVM-VTH	*Escherichia coli*	bla_{NDM}	>=32 (R)	>=32 (R)	≥128 (R)	≥64 (R)	≥64 (R)	≥64 (R)	≥64 (R)	≥64 (R)	≥8 (R)	≥16 (R)	2 (S)	≥16 (R)	≥4 (R)	≤16 (S)	64 (R)	≥64 (R)	>=320(R)
314530	KSVM-VTH	*Escherichia coli*	bla_{NDM}	>=32 (R)	>=32 (R)	≥128 (R)	≥64 (R)	≥64 (R)	≥64 (R)	≥64 (R)	≥64 (R)	≥8 (R)	≥16 (R)	2 (S)	≥16 (R)	≥4 (R)	≤16 (S)	≤16 (S)	≥64 (R)	>=320(R)
325491	KSVM-VTH	*Escherichia coli*	bla_{NDM}	>=32 (R)	>=32 (R)	≥128 (R)	≥64 (R)	≥64 (R)	≥64 (R)	≥64 (R)	≥64 (R)	≥8 (R)	8 (R)	≥64 (R)	≥16 (R)	≥4 (R)	≤16 (S)	64 (R)	16 (R)	>=320(R)
304853	KSVM-VTH	*Escherichia coli*	$bla_{OXA-48-like}$	>=32 (R)	>=32 (R)	≥128 (R)	≥64 (R)	16 (R)	≥64 (R)	8 (R)	32 (R)	2 (R)	1 (S)	2 (S)	≤1 (S)	≥4 (R)	≤16 (S)	64 (R)	≥64 (R)	>=320(R)

Table S2. Genomic characteristics, sequence types, antibiotic resistance gene (ARG) profiles, and plasmid replicon types of the nine whole-genome sequenced *Escherichia coli* isolates.

						ARG profile									
Sample number	BioSample Accession	Genome accession	Sequence Type	ST clonal complex	*E. coli* Phylogroup	Beta lactams	Fluoroquinolone	Tetracycline	Aminoglycoside	Macrolide	Sulfonamide	Trimethoprim	Chloramphenicol	Other Classes	Plasmid replicons
**313942**	SAMN56009510		38	ST38 Cplx	D	blaTEM; blaCTX-M-15	gyrA_D87N;gyrA_S83L;parC_S57T;parC_S80I;parE_S458A	tet(A)	aadA2;aac(3)-Ile;aadA1	-	-	dfrA51;dfrA1	-	Fosmidomycin:cyaA_S352T;Lincosamide:lnu(F);Streptothricin:sat2	IncFIB, IncFIC, IncX1_4, Col, p0111
**304137**	SAMN56009511		617	ST10 Cplx	A	blaTEM; blaCTX-M-14; blaDHA-1	gyrA_D87N;gyrA_S83L;parE_S458A;parC_S80I;qnrB4	tet(A);tet(M)	aadA1;ant(2")-Ia;aadA2	mph(A)	sul2;sul1	dfrA7;dfrA12	cmlA1;floR	Nitrofuran:nfsA_G126R;Lincosamide/Macrolide/Streptogramin:erm(B)	IncFIB, IncFIC, IncFII (pHN7A8)
**299962**	SAMN56009512	JBVUBI000000000	5699	-	A	blaTEM-1; blaCTX-M-55	gyrA_D87N;gyrA_S83L;parC_S80I	tet(A);tet(B)	aadA1;aph(3")-Ib;aph(6)-Id;aadA2;aac(3)-IId	mph(A)	sul2;sul1	dfrA12	floR;catA1	-	IncFII, IncI1, IncX1_4, Col
**298960**	SAMN56009513	JBVUBH000000000	5529	-	A	blaTEM-176; blaOXA-1; blaCTX-M-15	aac(6')-Ib-cr5;gyrA_D87N;gyrA_S83L;parC_S80I;parE_S458A	tet(B)	aac(6')-Ib-cr5;aph(3')-Ia;aadA1;aadA5;aac(3)-IIe	mph(A)	sul3;sul1	dfrA17	catB3;floR	Nitrofuran:nfsA_G126R	IncFIA, IncFIB, IncFIC, IncX1, Col
**1354**	SAMN56009514	JBVUBG000000000	101	ST101 Cplx	B1	blaTEM; blaCTX-M-15	qnrS1	-	aadA1	mph(A)	-	-	-	-	IncB/O/K/Z_2, IncFII, Col
**970**	SAMN56009515	JBVUBF000000000	69	ST69 Cplx	D	blaCARB-2; blaCTX-M-55	-	tet(B)	aadA1;aadA2	-	sul3	dfrA16	cmlA1	Fosmidomycin:cyaA_S352T	IncFIA, IncFIB, IncFII, IncI1, IncR, Col
**936**	SAMN56009516	JBVUBE000000000	648	ST648 Cplx	F	blaOXA-1; blaCTX-M-15	gyrA_D87N;gyrA_S83L;aac(6')-Ib-cr5;parC_S80I;parE_S458A	tet(B)	aadA5;aac(6')-Ib-cr5	mph(A)	sul1	dfrA17	catB3	Fosmidomycin:cyaA_S352T, Rifamycin: arr	IncFIA, IncFIB, IncFII, Col
**922**	SAMN56009517	JBVUBD000000000	1431	-	B1	blaTEM-1; blaCTX-M-15	parC_S80I;parE_S458A;gyrA_D87N;gyrA_S83L	tet(A)	aph(3')-IIa;aph(3')-Ia;aph(3")-Ib;aph(6)-Id;aadA1;aadA2;rmtB1	mph(A)	sul3	dfrA51;dfrA14	floR;cmlA1	-	IncFIB, IncN, IncX1, Col
**845**	SAMN56009518		155	ST155 Cplx	B1	blaTEM-1; blaCTX-M-15	qnrS1	-	aph(3')-IIa;aph(6)-Id;aph(3")-Ib;aph(3')-Ia;rmtB1	-	sul2	dfrA14	floR	Bleomycin:ble	IncHI2, IncHI2A, IncI1, IncN, IncX1, IncY

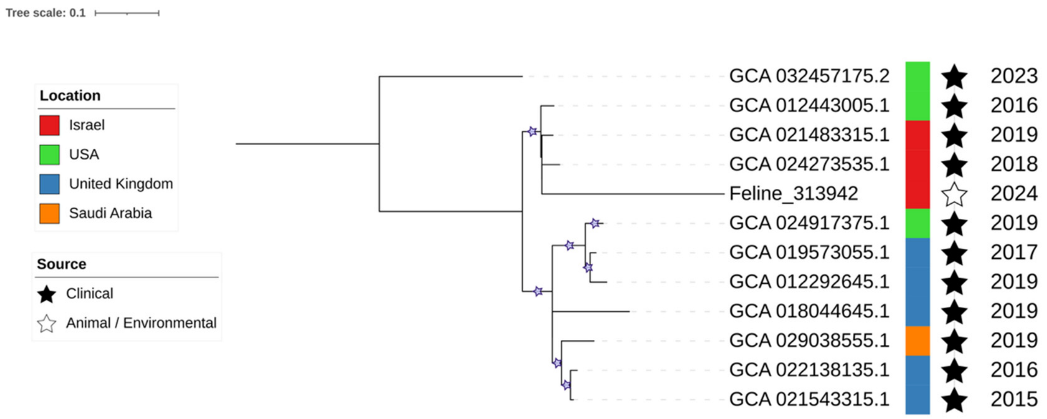

Figure S1. Maximum likelihood phylogenetic tree of ST38 genome assemblies based on core genome single nucleotide polymorphisms (SNPs). Tip labels are annotated with source, country and collection year. The tree is midpoint-rooted. Bootstrap support values > 90 are indicated by purple stars. For ST155, the phylogeny placed our isolate Feline_845 in a cluster with isolates from Japan (2017) and the USA (1979), with pairwise core SNP distances of 107–133.

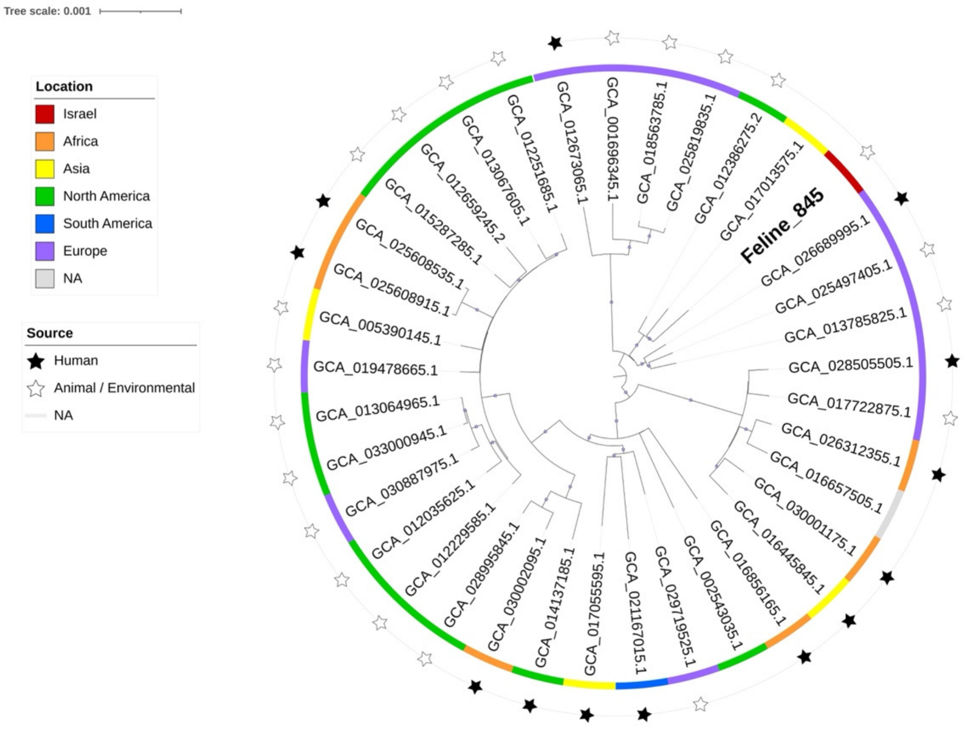

Figure S2. Maximum likelihood phylogenetic tree of ST155 genome assemblies based on core genome SNPs, annotated with country and sample source. The tree is midpoint-rooted. Bootstrap support values > 90 are indicated by purple stars.

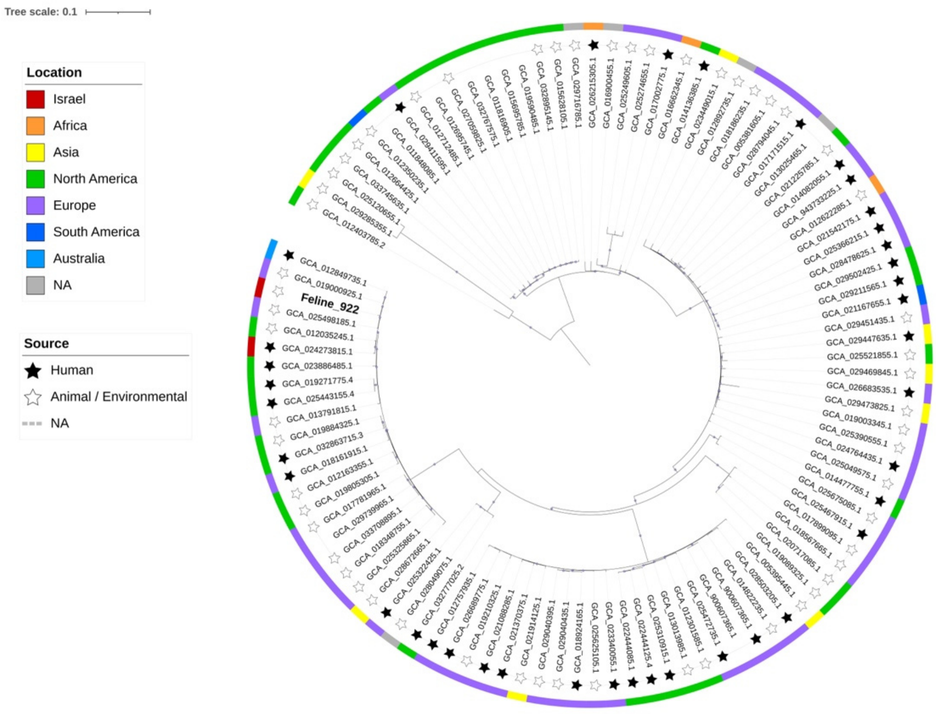

Figure S3. Maximum likelihood phylogenetic tree of the ST1431 genome assemblies, based on core genome single nucleotide polymorphism (SNP), with sample source, country and collection year. Tree was rooted at midpoint. Bootstrap values >90 are presented by purple stars.
